# Glutamate Systems in DSM-5 Anxiety Disorders: Their Role and a Review of Glutamate and GABA Psychopharmacology

**DOI:** 10.3389/fpsyt.2020.548505

**Published:** 2020-11-19

**Authors:** Madeeha Nasir, Daniel Trujillo, Jessica Levine, Jennifer B. Dwyer, Zachary W. Rupp, Michael H. Bloch

**Affiliations:** ^1^Yale Child Study Center, Yale University School of Medicine, New Haven, CT, United States; ^2^Yale Department of Radiology and Biomedical Imaging, Yale University School of Medicine, New Haven, CT, United States; ^3^Frank H. Netter School of Medicine, Quinnipiac University, North Haven, CT, United States; ^4^Yale Department of Psychiatry, Yale University School of Medicine, New Haven, CT, United States

**Keywords:** glutamate, anxiety, pharmacology, psychiatry, clinical trials, preclinical trials

## Abstract

Serotonin reuptake inhibitors and benzodiazepines are evidence-based pharmacological treatments for Anxiety Disorders targeting serotonin and GABAergic systems, respectively. Although clearly effective, these medications fail to improve anxiety symptoms in a significant proportion of patients. New insights into the glutamate system have directed attention toward drugs that modulate glutamate as potential alternative treatments for anxiety disorders. Here we summarize the current understanding of the potential role of glutamate neurotransmission in anxiety disorders and highlight specific glutamate receptors that are potential targets for novel anxiety disorder treatments. We also review clinical trials of medications targeting the glutamate system in DSM-5 anxiety disorders. Understanding the role of the glutamate system in the pathophysiology of anxiety disorder may aid in developing novel pharmacological agents that are effective in treating anxiety disorders.

## Key Points

- Serotonin Reuptake Inhibitors and benzodiazepines are evidence-based pharmacological treatments for Anxiety Disorders. These primarily target serotonin and GABA systems, respectively.
- Although clearly effective, these medications do not help improve anxiety symptoms in many patients and have significant adverse effects.
- Preclinical studies and H-MRS studies show anxiolytic potential in targeting glutamate systems.
- While some clinical studies have shown some promise, most have been largely underpowered or not used placebo groups. Larger placebo-controlled trials need to be conducted in order to verify their utility in the management of anxiety disorders.

## Introduction

Anxiety disorders affect ~18% of adults making them one of the most common psychiatric disorders (1). The Diagnostic Statistical Manual 5 (DSM-5) lists separation anxiety, generalized anxiety disorder (GAD), social anxiety disorder (SAD), specific phobia, panic disorder, and agoraphobia under the anxiety disorders (2). While the DSM-5 recently removed obsessive-compulsive disorder (OCD) and post-traumatic disorder (PTSD) from the category of anxiety disorders, anxiety is still considered a prominent and distressing symptom in these and other psychiatric disorders such as autism spectrum disorders (ASD) and depression. Co-morbidity of anxiety disorders with other psychiatric disorders is associated with a more chronic, treatment resistant course and with elevated suicide risk (3, 4). Additionally, patients with anxiety disorders have increased risk of substance abuse and certain medical disorders such as hypertension and irritable bowel syndrome (5). Thus, anxiety's link to impairment is well established across a broad constellation of domains and disorders. The high prevalence of anxiety as a primary or comorbid disorder and its significant functional disability result in a high personal, social and economic cost (6).

Given the high impairment and cost of anxiety disorders, an array of psychotherapeutic approaches and medications have been assessed and validated for the treatment of anxiety disorders. Both pharmacological and psychotherapeutic treatments are currently the recommended first-line treatments for DSM-5 anxiety disorders (7–9). The combination of pharmacological and behavioral treatments for anxiety disorders is likely more effective than either intervention alone, especially in patients with moderate-to-severe anxiety symptoms (10, 11). Cognitive behavioral therapy is currently the recommended first-line psychotherapeutic treatment for DSM-5 anxiety disorders (7–9), although several other psychotherapeutic approaches have some demonstrated efficacy in the treatment of anxiety disorders (10). However, access and successful dissemination of CBT and other psychotherapies remains a major treatment challenge. Furthermore, 46.4% of the patients who are lucky enough to have access to gold-standard psychotherapeutic treatment for anxiety disorders, do not respond to therapy (12). Others still have substantial residual anxiety despite having a good response to therapy, or are resistant to engaging in therapy due to concerns about cost, time, stigma or fear of engaging in exposures (13). Due to these reasons, pharmacological treatments remains integral to the care of patients with anxiety disorders, despite the fact that psychotherapeutic treatments are as or more effective than any pharmacological treatment for anxiety currently available (10).

First-line pharmacological treatment for DSM-5 anxiety disorders primarily consists of antidepressants that modulate serotonin neurotransmission such as selective serotonin reuptake inhibitors (SSRIs) and serotonin norepinephrine reuptake inhibitors (SNRIs) (10). Both SSRI and SNRI medications have demonstrated significant benefits compared to placebo in treating anxiety disorders in meta-analysis of randomized controlled trials (RCTs) (10). Although current treatment guidelines recommend treating patients for at least 8 weeks at the maximally tolerated dose of SRI medication, longitudinal meta-analysis found that it takes only four weeks for SSRIs (SMD = 0.60) and two weeks for SNRIs (SMD = 0.51) to attain a statistical significant benefit compared with placebo (14). However, the maximal treatment benefit of SSRI and SNRI medications for anxiety is fully observed until 12 weeks after the initiation of treatment (14). This delay in therapeutic benefit of serotonergic antidepressants can be exacerbated in clinical practice where dose titration is typically more conservative than that adhered to in clinical trials (14, 15). Serotonergic antidepressants are also associated with significant sexual side-effects that can limit tolerability and impact adherence (16–19). Although typically quite well-tolerated, serotonergic antidepressants are associated with other common adverse effects include nausea, diarrhea, diaphoresis, headaches, tremor, asthenia, insomnia, and somnolence (20–25). Finally, sudden discontinuation of these serotonergic antidepressants can result in a withdrawal syndrome which can include worsening of anxiety symptoms, panic attacks, dizziness, nausea that can be quite substantial and may take several few weeks to resolve without treatment (26). In summation, serotonergic antidepressants are only partially effective (leaving many patients who do not respond or only partially respond to treatment) and when beneficial take a long time to become fully effective and have a slew of adverse effects.

Benzodiazepines are another evidence-based pharmacological treatment that are commonly utilized and have strong evidence of efficacy for the treatment of DSM-5 anxiety disorders. Benzodiazepines act as positive allosteric modulators at y-aminobutyric acid (GABA)-A receptors, enhancing overall GABAergic activity. GABA functions as the primary inhibitory neurotransmitter in the central nervous system. Benzodiazepines can rapidly improve anxiety symptoms, unlike the serotonergic antidepressants, which take more time for benefits to accrue. However, benzodiazepines have the potential for abuse and dependence and are associated with greater adverse effects than serotonergic antidepressants. Common side-effects associated with acute benzodiazepine treatment include ataxia or incoordination, fatigue, slurred speech, drowsiness, sexual dysfunction (decreased libido, or anorgasmia), xerostomia, constipation, and light-headedness (27–29). Long-term use of benzodiazepines can be associated with dependency (and thus reduced efficacy at a given dose), rebound anxiety, a discontinuation syndrome and memory impairment (15). Therefore, although quite effective in the acute treatment of anxiety disorders, benzodiazepine use is often reserved for as needed short-term relief on anxiety symptoms, in patients refractory to serotonergic antidepressants and psychotherapy, and as an adjunct when starting serotonergic antidepressants to bridge the gap of their “therapeutic delay” and prevent worsening of anxiety at the beginning of treatment (15).

Although there exist several effective psychotherapeutic and pharmacological treatments currently in our armamentarium for the treatment of DSM-5 anxiety disorders, there still exists a substantial need for novel treatments, as currently available treatments either do not help a substantial minority of patients or often leave substantial residual symptoms. Additionally, they have bothersome side-effects and typically take several months to reach full efficacy.

Here we will review the current evidence-base of pharmacotherapies that target glutamate and GABA neurotransmission for the treatment of DSM-5 anxiety disorders. Glutamate systems in corticolimbic circuits interact with GABAergic, serotonin, dopamine, and other systems involved in the stress response, which implies a potential role in the circuitry underlying anxiety disorders (30–32). Consistent with this hypothesized role, investigational drugs that target glutamate receptors have shown promise as treatment strategies for anxiety disorders (33–35). This review aims to summarize the characteristics and potential pharmacological targets of the glutamate system. In addition, clinical trials of the main glutamatergic drugs for anxiety disorders as classified under the DSM-5 will be reviewed.

## The Glutamate System

**Figure 1:**
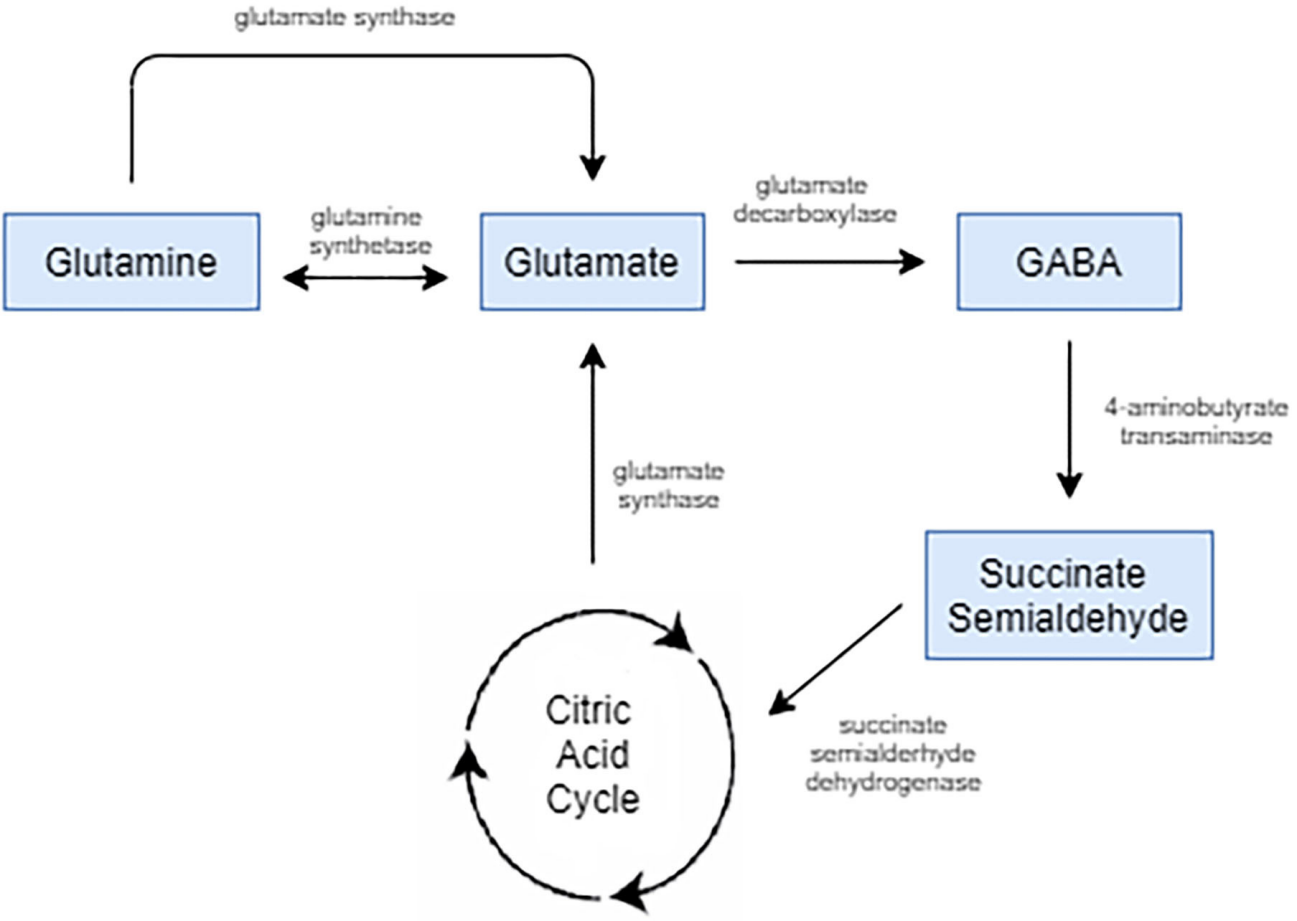
Glutamate is the biological precursor for GABA. Glutamate is synthesized from the nonessential amino acid glutamine, and glutamate is converted into GABA by the enzyme glutamate decarboxylase.

Glutamate is the chief excitatory neurotransmitter in the mature central nervous system (CNS). Its projections take part in effectively all neural circuits, interconnecting cortical, and subcortical systems (36). It is prevalent throughout the CNS and has been found to play important physiological roles in neurodevelopment, cognition, and learning and memory formation (37, 38). It plays a part in a variety of cellular activities in both neurons and glia beyond glutaminergic neurotransmission. It has a role in protein synthesis, energy metabolism, as a part of the tricarboxylic acid cycle, is a precursor for both glutathione, which mitigates oxidative stress, and GABA, an inhibitory neurotransmitter as depicted by Figure 1 (39).

Glutamate has the characteristics of a typical neurotransmitter (36). It is synthesized and packed into vesicles at pre-synaptic terminals of glutamatergic neurons, by vesicular glutamate transporters (vGluT) (40). On arrival of an action potential, the vesicles fuse with the pre-synaptic membrane and release glutamate into the synaptic cleft. Once released, glutamate binds with both the postsynaptic receptors, which transmit the signal onto the next neuron, and presynaptic receptors, which prevent further release of glutamate resulting in negative feedback.

Glutamate receptors can either be ionotropic receptors, which are ligand gated cation channels, or metabotropic receptors, which are G-protein coupled transmembrane proteins. Ionotropic receptors are of three types: N-methyl-D-aspartate (NMDA), α-amino-3-hydroxy-5-methyl-4-isoxazole propionic acid (AMPA) and kainite receptors. Metabotropic receptors have eight types termed mGluR_1−8_ (41).

Heteromeric NMDA receptors are present in high densities in the hippocampal region, frontal cortex and other limbic areas of the brain. They are comprised of two conserved NR1 subunits and two or three regionally specific NR2 subunits. Activation requires three conditions: occupation of glutamate binding site on the NR2 subunit, occupation of the co-agonist glycine_B_ (GLY_B_) site on the NR1 subunit, and membrane depolarization (30). Thus, both pre- and post-synaptic neurons need to be depolarized in order to activate NMDA receptors, adding an additional level of regulation to glutamate transmission (42). Once activated, a Mg^2+^ ion that blocks the central pore is released, allowing influx of Na^+^, and importantly, Ca^2+^ into the cell, which plays a crucial part in synaptic plasticity and memory formation (43).

In contrast, the channels on the AMPA receptors open when glutamate molecules bind onto a minimum of two out of four available binding sites, allowing influx of Na^+^, K^+^, and Ca^2+^ ions (44). While similar in structure, kainate receptors open for a shorter time than AMPA receptors. Unlike the NMDA and AMPA receptors which transmit the action potential, kainate receptors are believed to act as pre- and post-synaptic modulators (45).

mGLuR are transmembrane receptors, coupled to G-proteins, that when activated by an extracellular ligand trigger intracellular cascades that modulate neuronal activity. Different subtypes are expressed differentially in specific brain areas. For example, mGlu_1_ and mGlu_5_ are predominantly in the amygdala, hippocampus and thalamus, whereas mGlu_3_ is located primarily on glia (38).

**Figure 2:**
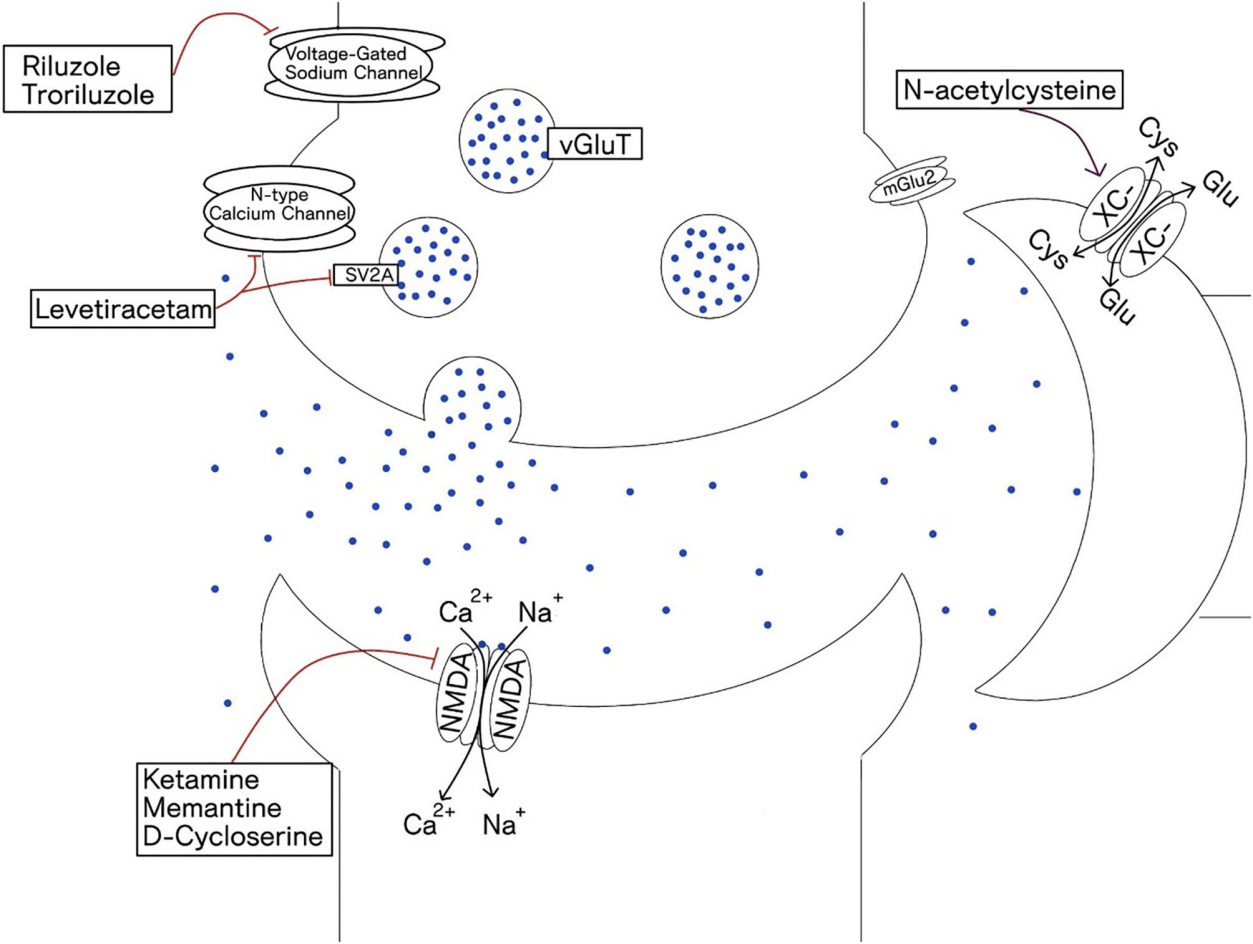
Depicts glutaminergic synapse and the receptors that are the site of action of glutaminergic drugs. Glutamate is packed into vesicles by vesicular glutamate transporter (vGluT). It binds to both ionotropic receptors and metabotropic receptors. Glial cells play a primary role in glutamate reuptake thus terminating the glutamate synaptic signal. Steady-state extra synaptic glutamate levels are also regulated by the glial cystine-glutamate antiporter (XC-).

It is not only the receptor subtype that determines function within a circuit, but also the location of those receptors. Extra-synaptic glutamate receptors have all together distinct effects compared to synaptic receptors. For example, while synaptic NMDA receptor activation transmits action potentials across neurons and promotes neuronal growth and plasticity, extra-synaptic NMDA receptors inhibit these processes and can result in neuronal damage (46). Significant physiologic energy is devoted to containing glutamate within the synapse with tight regulation of spill over via high affinity glutamate transporters, primarily on glial cells (mainly astrocytes). Activation of extra-synaptic receptors occurs when these reuptake mechanisms are overwhelmed and contributes to excitotoxic cascades and cell death (47). Table 1 gives a list of drugs acting on the glutamate system based on their mechanisms of action and Figure 2 depicts a glutamatergic synapse and displays the receptor sites at which the agents discussed in this review act.

**Table 1:** Describes the mechanisms with which drugs affect glutamate neurotransmission.

**Mechanism of action**	**Drug**	**Comment**
NMDA antagonism	Ketamine	Non-competitive antagonist of NMDA receptors.
	Memantine	Non-competitive antagonist of NMDA receptors.
	D-Cycloserine	At low concentrations, acts as a partial NDMA agonist, but demonstrates antagonistic effects in higher concentrations.
↓ Synaptic glutamate release	N-Acetylcysteine	Increases extracellular glutamate via its action on glial cystine-glutamate antiporters; free glutamate then activates mGluR2/3 on the presynaptic nerve terminals and reduces synaptic glutamate release via negative feedback.
	Riluzole	Blocks presynaptic voltage-gated sodium and calcium channels inhibiting glutamate release; potentiates glial reuptake of glutamate; modulates trophic and toxic effects of glutamate
	Gabapentinoids	Inhibit central nervous system voltage-gated α2-δ calcium channels, leading to decreased glutamate release.
↑ GABA concentration	Levetiracetam	Binds to the synaptic vesicle protein SVA2, interfering with neurotransmitter exocytosis, and via inhibition of N-type calcium channels and increases GABA concentration
	Valproic acid	Blocks voltage-gated Na^+^ channels and increases GABA concentrations by reducing its degradation.
	Tiagabine	Selectively inhibits GABA reuptake.
AMPA/kainite receptor agonist	Topiramate	Regulates Na+ and Ca2+ channel opening, potentiates GABA, and acts as an antagonist on AMPA and kainate receptors

## Role of Glutamate in Anxiety Disorders

### Proton Magnetic Resonance Spectroscopy (H-MRS) Studies of Glutamate in Anxiety Disorders

H-MRS is an imaging technique that quantifies endogenous brain metabolites *in vivo*. Most previous H-MRS studies in anxiety disorders have focused on the CNS specific metabolite, N-acetylaspartate (NAA), since it has a prominent signal in MRS with levels correlating to neuronal integrity (48–52). Some studies, however, have investigated region specific changes in glutamate levels in patients with anxiety disorders.

Glutamate (Glu) and glutamine (Gln) have similar molecular structures which give rise to similar magnetic spectra and thus have traditionally been considered to constitute one pool denoted Glx. Glx levels have been found to be higher (53) or unchanged in OCD (54) and lower in social anxiety disorder (55). These inconsistent results could be due to a variety of different reasons. For example, measuring Glx levels does not aid in studying them separately or in conditions where Glu and Gln concentrations are altered in opposing directions. Another complication is that since glutamate has multiple roles outside of neurotransmission, it is difficult to distinguish between an increase in extracellular glutamate seen due to microdialysis vs. an increase in synaptic glutamate release. Additionally, imaging studies often suffer from low power and familywise error due multiple inferential statistical tests done on the same dataset, resulting in a high rate of type 1 errors, variation within studies, and uncertainty. The studies reviewed here are pilot studies and are to the same limitations and thus require larger multisite replications.

Newer MRS techniques can now distinguish between Glu and Gln peaks, which has allowed rapid increase in our understanding of the dynamics between Glu and Gln (56). Higher frontal cortex Glu levels have been detected in healthy subjects with high trait state anxiety compared to low trait state anxiety (57). Patients with social anxiety were found to have a 13.2% higher Glu levels in the anterior cingulate cortex (ACC) compared to controls. This increase was correlated with the severity of social anxiety symptoms (58).

### Role of Glutamate in Anxiety-Related Disorders

Glutamate has been implicated in depression, OCD and PTSD (33, 59, 60), which have overlapping symptomatology or are often comorbid with anxiety disorders. Post-mortem studies on patients with depression have found altered glutamate levels in plasma, cerebrospinal fluid and brain tissue as well as differences in NMDA receptor expression and affinities (61). Blood glutamate scavengers, such as oxaloacetate and pyruvate inactivate glutamate by converting it into 2-ketoglutarate leading to a decrease in glutamate blood levels. This decrease in blood levels causes glutamate to shift down its concentration gradient from the brain to the blood resulting in lower brain glutamate levels. Animal studies have shown potential benefits in the treatment of post-stroke depression (62). Genetics studies also suggest links between genes associated with glutamatergic transmission and depression (63, 64). Likewise, OCD patients have shown aberrant levels of glutamate in cerebrospinal fluid (60, 65). Like in depression, there have been some findings of elevated Glx levels in OCD patients, however, they have also been inconsistent (42, 66, 67). While there have been associations between several genes related to the glutamatergic system and OCD, they too have not been consistent. The best replicated finding is the correlation of OCD risk with variants of the glutamate transporter EAAT3, coded by *SLC1A1* gene (68–75). The causative mutations and their effects have not been elucidated (42). Variants of this gene were also associated with greater risk of PTSD after trauma and greater symptom severity (64). Another study with PTSD patients also found increased Glx levels in the rostral ACC when compared to healthy controls and those in remission (76). Finally, medications that target the glutamate system have been utilized to treat these disorders with some success, thus, reinforcing the argument for investigating glutamatergic agents for treatment of anxiety disorders (42, 59, 61).

### Preclinical Studies of Glutamate in Anxiety Disorders

Preclinical studies have provided a significant scientific rationale for the potential of glutamate modulators in the management of anxiety disorders (30, 33, 77). Stress is a key factor in the development of anxiety disorders and this is simulated using a variety of animal stress models. Stressing a rat has been shown to stimulate glutamate release in the prefrontal cortex of the its brain (78, 79). In contrast to acute stress which has shown to increase glutaminergic transmission in the prefrontal cortex and other limbic regions, chronic stress has been associated with a decrease in glutamate receptors resulting in lower glutamate transmission (80). The glutamate system also plays a major role in the extinction process in fear learning and extinction paradigms (81). As discussed below, the results of stress and fear studies on animal models are in line with this theory (82).

Animal models do not reflect all the complexities of specific anxiety disorders instead, they aim to create a state of anxiety-like behavior that can be generalized to these disorders (83). Unconditioned anxiety models rely on creating situations where the rats face opposite motivational forces to explore or to hide in novel situations (e.g., the elevated plus maze and the social interaction test) or can be predator based (e.g., cat and rat exposure test). Animal anxiety models can also involve classical conditioning (e.g., the fear-potentiated startle response and place aversion test) or operant conditioning (e.g., Geller-Seifter test and Vogel conflict test) (84). Finally, there are pathophysiological models which utilize chronic immobility or stress and trauma paradigms (84, 85).

These models have been used to assess the anxiolytic activity of drugs acting on NMDA, AMPA, kainite, and mGLuR receptors. Injecting the NMDA receptor blocker, DL-2 amino-5-phosphonopentanoic acid (AP5), into the pontine reticular nucleus of rates attenuated the fear potentiated startle response in a dose dependent manner (86). When injected into the amygdala, it inhibited the acquisition of the fear potentiated startle response, but not the expression of previously acquired fear responses (87).

The effects of ketamine (an NMDA antagonist) on anxiety have also been studied in rat models, with one finding no significant differences in anxiety levels as measured by the elevated plus maze test between rats exposed to subanesthetic ketamine doses (30 mg/kg) and saline-injected controls (88); another study found that a single anesthetic ketamine dose (100 mg/kg) caused rats to exhibit higher anxiety as measured by performance in the open field test (89). In another study, the systemic administration of intra-amygdala infusions of D-Cycloserine (DCS), a partial NMDA receptor agonist that can antagonize the NMDA receptor at high doses (90) resulted in the dose dependent facilitation of fear extinction (91–94). Thus, NMDA blockade in rats via administration of different pharmacological agents has demonstrated mixed effects on anxiety levels, with more studies required to elucidate the cause of these differences.

Both kainic acid and topiramate are AMPA/kainite receptor agonists and have shown to decrease the fear potentiated startle response and stress induced startles responses in rats (95, 96). Administrating methyl-6-(phenlythynyl)-pyridine (MPEP), a mGluR_5_ antagonist, resulted in decreased in fear potentiated startle (97) and increased punished responding (decreased avoidance of painful shock in order to obtain reward) (98). Unlike mGluR_5_ which are postsynaptic at the glutamatergic synapse and coupled with G_q_-proteins, mGluR_2/3_ exist at the presynaptic end and are coupled with inhibitory G_i_/G_o_ proteins (41). The anxiolytic effect of activating these receptors is seen across several rat anxiety models, after systemic and oral administration of LY354740, a mGluR_2/3_ agonist, resulting in decreased fear potentiated startle responses (99, 100), decreased lactate-induced panic (101) and an increase exploration time in the exposed arms of an elevated plus maze (102). Finally, lamotrigine which inhibits glutamate response by blocking sodium channels and has shown to decrease postsynaptic NMDA receptor mediated excitatory postsynaptic potential in rat amygdala neurons (103). Similarly, rilozule also blocks voltage gated sodium channels, in addition to blocking excitatory amino acid receptors and various calcium channels (103). When injected in rates, both these drugs showed anxiolysis and increased conditioned emotional response rates during the presentation of food with a light which was previously paired with shock (103). Taken together these data indicate a general trend toward activation of the glutamate system and fear startle response acquisition and its blockade with facilitating fear response extinction and anxiolytic-like effect. However, this may not be a universal rule as it is contradicted by the fact that AMPA/kainite receptor agonists reduced potentiated startle response. The mixed pharmacology of most drugs makes it challenging to solely attribute the anxiolysis to one target. Additionally, alternative justifications for this anxiolytic-like effect cannot be ruled out, for example, that it instead is the result of drug impaired memory and attention.

### Clinical Studies of Glutamate Psychopharmacology in Anxiety Disorders

This decade has seen increasing support for the utilization of glutamate modulating drugs in psychiatric conditions. Medications already in clinical use for conditions like epilepsy, neurodegenerative diseases, and alcohol abuse were found to have glutamatergic mechanisms of actions (39). The FDA approved indications of the drugs are listed in Table 2. There have been several trials to test their use as treatments for anxiety disorders (see Table 3).

**Table 2:** Lists the FDA approved indications of the agents and the anxiety disorders they have been tested for.

**Drug**	**FDA indications**	**Anxiety disorder**	**Dose range, mg/d**
Ketamine	Induction and maintenance of anesthesia	Social anxiety disorder, GAD	Ascending doses of 0.25, 0.5, 1.0 mg/kg at weekly intervals
Memantine	Moderate-severe dementia of the Alzheimer's type	GAD	10–20 BID
D-Cycloserine	Tuberculosis, urinary tract infection	Social anxiety disorder, Acrophobia	50 before exposure therapy
N-Acetylcysteine	Acetaminophen toxicity; mucolytic agent for inflammatory pulmonary conditions	GAD; Social anxiety disorder	1200–3000 BID
Riluzole	Amyotrophic lateral sclerosis	GAD	100–200
Pregabalin	Diabetic neuropathic pain, Post-herpetic neuralgia, epilepsy	GAD, Social anxiety disorder	150–600 BID
Gabapentin	Epilepsy, Post-herpetic neuralgia	Social anxiety disorder	900–3600 TID
Levetiracetam	Epilepsy	Social anxiety disorder	1000–3000 BID
Tiagabine	Epilepsy	GAD	16–32 BID
Topiramate	Epilepsy, migraine	Social anxiety disorder	25–400
Valproic acid	Mania, epilepsy, Migraine	Social anxiety Disorder	750–2500 TID

*Disorders: GAD, Generalized Anxiety Disorder*.

**Table 3:** Summary of glutamate psychopharmacology trials for anxiety disorders.

**References**	**Medication**	**Indication**	**Average Dose**	**Dose**	**Sample Size**	**Design**	**Duration (weeks)**	**Measures**	**Results**
**Glutamatergic**									
Glue et al. (104)	Ketamine	GAD/SAD	0.58 mg/kg	Ascending doses of 0.25, 0.5, 1.0 mg/kg at weekly intervals	12	Ascending single-dose, uncontrolled, open label study	3	FQ and HAM-A	Dose dependent improvement in anxiety symptoms. 10 of 12 responded at 0.5 and/or 1 mg/kg doses
Taylor et al. (105)	Ketamine	SAD	0.5 mg/kg	0.5 mg/kg over 40 min	18	Double-blind, randomized, placebo-controlled crossover trial	2 week follow up after 1 dose	LSAS, VAS-Anxiety	Significant improvement in anxiety symptoms measured 1 day-2 weeks following treatment
Hofmann et al. (106)	DCS	SAD	50 mg	50 mg 1 h prior to therapy	27	Randomized, double-blind, placebo controlled trial of DCS as adjunctive treatment to exposure therapy	5	SPAI, LSAS	Less social anxiety after treatment and at 1 month follow-up
Guastella et al. (107)	DCS	SAD	50 mg	50 mg before each treatment	56	Randomized, double-blind, placebo controlled trial of DCS as adjunctive treatment to exposure therapy	5	SPAI, LSAS, BFNE	Reduced social anxiety when DCS is given before therapy.
Mathew et al. (108)	Riluzole	GAD	100 mg	100 mg/d	15	Open label trial	8	HAM-A	Clinically significant reduction of generalized anxiety after treatment
**GABAergic**									
Simon et al. (109)	Levetiracetam	SAD	2013 ± 947.5 mg	250 mg/d for first week and titrated up to a maximum of 3000 mg/d	20	8 week open label flexible dose study	8	LSAS, HAM-A	Clinically significant reduction in social anxiety after treatment as found in two scales
Zhang et al. (110)	Levetiracetam	SAD	2279 mg	Started at 500 mg/day for 4 days increased at the rate of 500 mg every 3–4 days, to 2000 mg/day by day 14, upto maximum dose of 3000 mg (1500 mg BID).	18	Double blind Randomized control trial	7	MINI, BSPS, LSAS, SPIN	No significant findings
Stein et al. (111)	Levetiracetam	SAD	1180+/- 780 mg	Started at 250 mg/d and flexibly titrated up to maximum dose of 3,000 mg/d (1,500 mg bid)	217	Double blind randomized controlled trial	12	LSAS	No significant findings
Feltner et al. (112)	Pregabalin	GAD	50 or 200 mg	Started at 4 mg/d and flexibly dosed twice a day to a maximum of 16 mg/d	271	Double blind, fixed-dose, parallel group, randomized control trial	4	HAM-A	No significant findings
Pande et al. (113)	Pregabalin	GAD	?	150 mg/day (50 mg t.i.d.) and 600 mg/day (200 mg t.i.d.)	276	Randomized double blind controlled trial	4	HAM-A	Pregabalin significantly reduced the total HAM-A score compared with placebo.
Rickels et al. (114)	Pregabalin	GAD	300 or 450 or 600 mg	Started at 300 mg/d and depending on assigned dosage, titrated to 450 mg/d on day 4 and to 600 mg/d on day 7	454	Randomized double blind controlled trial	4	HAM-A	Significant reduction in symptoms of generalized symptoms with pregabalin compared to placebo
Montgomery et al. (115)	Pregabalin	GAD	400 or 600 mg	For 400 mg/day: Started at 100 mg/d, dose was doubled every 2 days until max dose of 400 mg/d. For 600 mg/d: started at 150 mg/d, 150 mg/d was added on every 2 days until max dose of 600 mg.	421	Randomized double blind controlled trial	6	HAM-A	Pregabalin reduced symptoms of generalized anxiety compared to placebo with improvement in HAM-A and other secondary measures.
Pande et al. (116)	Gabapentin	SAD	?	900 mg/d t.i.d. to 3,600 mg/d t.i.d.	69	Randomized, double-blind, placebo controlled flexible dose trial.	14	LSAS, BSPS, SPIN, MMFQ, HAM-A	Significant reduction in symptoms of social phobia when taking gabapentin vs. placebo.
Pande et al. (117)	Gabapentin	Panic Disorder	?	Flexibly dosed between 600 mg/d to maximum of 3,600 mg/d, then tapered over 7 days at week 8.	103	Randomized, double-blind, placebo controlled flexibly dosed trial	8	PAS	No significant findings in total N and in patients with PAS score <20. In those with PAS score > or = 20, the gabapentin group showed significant improvement in PAS scores compared to placebo.
Lavigne et al. (118)	Gabapentin	Anxiety symptoms in breast cancer survivors	300 or 900 mg	300 mg, 900 mg	420	Randomized, double-blind, placebo controlled fixed dose trial.	8	STAI	Significant reductions in anxiety were seen at week 4 and 8 on both doses compared to placebo. Little, if any, difference in anxiolytic effect between the doses.
Pollack et al. (119)	Tiagabine	GAD	10.2 mg	Started at 4 mg/d and flexibly dosed twice a day to a maximum of 16 mg/d	266	Randomized, double-blind, placebo controlled flexible dose trial	8	HAM-A and HADS	Tiagabine reduced symptoms of GAD according to the observed case and mixed models repeated-measures (MMRM) analyses but not the primary LOCF analysis.
Kinrys et al. (120)	Valproic Acid	SAD	?	500 mg-2500 mg	17	Single-blind, flexibly dosed trial with a placebo lead in	12	LSAS and HAM-A	Social anxiety symptoms as measured by the LSAS and CGI-I scores significantly improved with treatment.
Aliyev and Aliyev (121)	Valproic Acid	GAD	1500 mg	1500 mg t.i.d.	80	Randomized, double-blind, placebo controlled trial	6	HAM-A	Significant decrease in HAM-A scores and greater number of responders compared to the placebo group.

*Disorders: GAD, Generalized Anxiety Disorder; SAD, Social Anxiety Disorder. Measures: AAQ, Acceptance and Action Questionnaire; AAVQ, Acrophobia Questionnaire with Avoidance; ACQ, Agoraphobic Cognitions Questionnaire; ATHI, Attitudes Toward Heights Inventory; BFNE, Brief Fear of Negative Evaluation Scale; BSPS, Brief Social Phobia Scale; BSQ, Body Sensations Questionnaire; FQ, Fear Questionnaire; HADS, Hospital Anxiety and Depression Scale; HAM-A, Hamilton Anxiety Inventory; LSAS, Liebowitz Social Anxiety Scale; MINI, MINI International Neuropsychiatric Interview; MI, Mobility Inventory; MMFQ, Marks-Mathews' Fear Questionnaire; PAS, Panic and Agoraphobia Scale; SPAI, Social Phobia and Anxiety Inventory; SPIN, Social Phobia Inventory; STAI, Speilberger State-Trait Anxiety Inventory; VAS-Anxiety, Visual Analog Scale; ?, not reported*.

## Pharmacological Agents Targeting Glutamate Neurotransmission

### Ketamine

Originally used as a rapid-acting intravenous anesthetic, in the last 20 years ketamine has been the subject of much research for its potential use in a variety of psychiatric disorders. In contrast to the delayed therapeutic effects of antidepressants acting on serotonin receptors ketamine has a fast acting mechanism of action the specifics of which remain largely debated. Ketamine acts as a noncompetitive antagonist of NMDA receptors, thus inhibiting downstream neuronal activation pathways (122), although more complex glutamatergic actions have been recently identified (123). Subsequent AMPA receptor activation is suggested to play a role since after 24 h ketamine has been metabolized to a point where direct NMDA antagonism could no longer be an ongoing mechanism of action and non-ketamine NMDA receptor antagonists do not exhibit robust ketamine like antidepressant effects (124).

Ketamine's efficacy in depression and mood disorders was first demonstrated in 2000 (125), and has subsequently been demonstrated in multiple clinical trials. A recent meta-analysis that included 9 RCTs comparing one-time infusion of ketamine to placebo showed a rapid and significant reduction in depressive symptoms (126). Ketamine has also shown efficacy in PTSD, with a midazolam-controlled trial showing that a single dose of 0.5 mg/kg of ketamine improved symptoms as measured by Impact of Event Scale-Revised scores at 24 h post-infusion; (127, 128), and in OCD, with an RCT showing improvement in symptoms (as measured by Y-BOCS scores) after a single dose of 0.5 mg/g of ketamine compared to placebo (129).

More recently, ketamine has also begun to be studied in anxiety disorders (130). One small open-label trial looking at the efficacy of ketamine in treatment-refractory generalized anxiety and/or social anxiety disorders (104) found that ten out of twelve patients responded (i.e., achieved ≥50% reduction in HAM-A scores and/or Fear Questionnaire scores) after three ascending doses of once weekly subcutaneous ketamine (0.25, 0.5, and 1 mg/kg); a subsequent trial from the same group further supported the safety and efficacy of ketamine (1 mg/kg) for this same population with once or twice weekly subcutaneous administrations over a period of three months (131). Furthermore, a double-blind randomized controlled crossover study enrolled 18 patients with SAD and found intravenous ketamine (0.5 mg/kg) resulted in significantly greater response rate (as measured by a reduction in Liebowitz Social Anxiety Scale scores of at least 35%) in the first two weeks post-infusion compared to placebo (105). Midazolam-controlled, parallel-group trials, examining the efficacy of repeated dosing of ketamine (e.g., twice-weekly for 2–4 weeks) are needed to examine the sustained effects of ketamine on both anxiety disorders and OCD.

### Memantine

Memantine is commonly used in Alzheimer's disease to improve cognitive symptoms (132), and similar to ketamine, it acts as an antagonist of ionotropic NMDA receptors. The differences in therapeutic profile between ketamine and memantine may be partially explained by their differing affinity for NMDA receptor subunits, as well as differences in subunit expression among synaptic and extra-synaptic receptors, and throughout the brain (133).

Given the hypothesized role of glutamatergic hyperactivity in the expression of obsessive and anxiety symptoms, one study compared the efficacy of memantine for OCD vs. GAD via a 12 week uncontrolled trial of 10 mg BID; the OCD group experienced a mean 40.6% reduction in YBOCS scores, while the GAD group only saw a reduction of 22.4% in HARS scores, suggesting potentially that memantine may have larger treatment benefits in OCD vs. GAD (134). A recent meta-analysis including 8 trials involving 125 participants with OCD patients found that 20 mg daily memantine augmentation to SRI pharmacotherapy for OCD suggested that after at least 8 weeks memantine augmentation was associated with a 3.6-fold greater likelihood of treatment response than placebo and an average 11-point improvement in Y-BOCS score (135). However, much of the promising data regarding memantine in OCD involves uncontrolled trials or small, single-site trials that are highly prone to bias. Larger, adequately powered, multi-site trials are needed to truly measure the treatment effect of memantine in anxiety and OCD.

### D-Cycloserine (DCS)

DCS is an analog of D-alanine that inhibits enzymes involved in bacterial peptidoglycan formation, and it is approved for use in resistant tuberculosis and select urinary tract infections (136). However, unlike alanine –which demonstrates full agonistic effects on the glycine-binding site of NMDA receptors–, DCS acts as a partial agonist, demonstrating *in vivo* agonistic effects in low concentrations and antagonistic effects in higher concentrations; this may be partly due to its high affinity for NMDA receptor subtype NR1/NR2C (where it exerts agonistic action), and low affinity for subtypes NR1/NR2A and NR1/NR2B (where it is antagonistic) (137).

Studies looking into the use of DCS in anxiety disorders have mostly been focused on its use as an augmentative agent to behavioral therapy, to enhance fear extinction. One meta-analysis from 2017 which included 21 studies found a statistically significant advantage of DCS over placebo in the augmentation of exposure-based CBT for anxiety-related disorders (including anxiety, obsessive-compulsive, and posttraumatic stress disorders), but the overall effect size was small (138). Another meta-analysis from the same year included 23 studies and found the overall effect of DCS to be almost non-existent among patients with anxiety and obsessive-compulsive disorder; slightly larger effect sizes were found among social anxious patients (139). A more recent study from 2019 recruited 81 patients with social anxiety disorder, who underwent a fear conditioning and extinction paradigm measuring skin conductance response to conditioned stimuli and shock expectancy ratings; DCS was not found to have a moderating influence on study outcome measures and there was no effect on the retention of extinction learning under the study paradigm (140). In totality, these data suggest that DCS at best has minimal benefit as an augmentation agent to behavioral therapy in anxiety disorders.

### N-Acetylcysteine (NAC)

NAC has classically been used in the context of acetaminophen toxicity to reduce hepatic injury (141), as well as a mucolytic agent for inflammatory pulmonary conditions (142). More recently, evidence has emerged supporting the potential use of NAC in a variety of neuropsychiatric disorders, including addiction and substance use disorders (143), neurodegenerative diseases such as spinocerebellar degeneration, Parkinson's disease, and others (144), schizophrenia (145), stroke (146), traumatic brain injury (147), obsessive-compulsive and related disorders (148), and depressive disorders (149), among other pathologies (150). NAC is a precursor to glutathione, a major antioxidant in the body, and its therapeutic properties are thought to be due its role in restoring cellular and mitochondrial redox imbalance, and reducing subsequent inflammation often seen with in neuropsychiatric conditions (150, 151). Additionally, NAC causes dose-dependent increases in extracellular glutamate via its action on cystine-glutamate antiporters found on glial cells; free glutamate then activates mGluR2/3 on the presynaptic nerve terminals and ultimately reduces synaptic glutamate release via negative feedback (150, 151).

Despite some promising initial data suggesting possible benefits of NAC in anxiety-related disorders such as OCD and depressive disorders, research examining its efficacy in anxiety disorders is quite sparse. Clinical trials specifically looking at the effects of NAC on anxiety are lacking. One case report showed improvement on generalized anxiety disorder and social phobia symptoms after adjunctive NAC (600 mg BID) was added to sertraline monotherapy, with a further improvement seen after increasing to 1200 mg (152). Another 16-week trial evaluating the efficacy of 3,000 mg NAC augmentation in forty OCD patients found a significant (*p* = 0.02) decrease in the Beck Anxiety Inventory (BAI) compared to placebo (153). However, when anxiety symptoms were analyzed in placebo-controlled trials looking at major depressive disorder (154) and trichotillomania (155), no significant differences were found in anxiety scores as measured by Hamilton Anxiety Rating Scale (HAM-A). Evidence examining the effects of NAC is both sparse and mixed at best. Larger, multi-site trials with anxiety as the primary measure are needed to estimate the effects of NAC for the treatment of anxiety disorders.

### Riluzole

Riluzole was originally developed as an anticonvulsant, though it only has FDA approval for amyotrophic lateral sclerosis. It likely acts upon the glutamatergic system via multiple mechanisms including blockade of presynaptic voltage-gated sodium and calcium channels to block glutamate release, potentiating glial reuptake of glutamate, and modulating trophic and toxic effects of glutamate (156).

The potential use of riluzole has been studied for a variety of psychiatric disorders with mixed success (157). In the setting of treatment-refractory OCD, for example, one RCT found modest improvement in Y-BOCS scores, though results were not statistically significant (158). For generalized anxiety disorder, an eight-week open trial was conducted on the effects of 100 mg/d of riluzole on HAM-A scores, with 80% of patients responding to the treatment (reduction in scores by 50% or more); further, 53% of these patients met remission criteria, with HAM-A scores becoming equal to or <7 (108). There are currently ongoing, mutli-site trials examining the effects of troriluzole, a riluzole prodrug, in the treatment of generalized anxiety disorder (NCT03829241) and OCD (NCT03299166).

## Clinical Trials Targeting GABAergic Neurotransmission

In contrast to glutamate, GABA is the primary inhibitory neurotransmitter of the nervous system. Anxiety is thought to stem from an imbalance between the excitatory and inhibitory systems resulting in dysregulation (159). Thus, medications which increase GABA activity will counterbalance the excitatory action of glutamate resulting in anxiolysis (160). Several medications that modulate ionotropic (GABA_A_) and metabotropic (GABA_B_) receptors are widely used in the treatment of disorders such as anxiety, epilepsy, insomnia, spasticity (161). Benzodiazepines act on GABA_A_ receptors which are highly complex due to their structural heterogeneity, their many connected binding sites and the numerous chemically distinct ligands that can bind to them (161). Thus, there is a growing interest in the development and application of subtype-selective drugs that will achieve specific therapeutic benefits without undesirable side effects.

### Levetiracetam

Levetiracetam is an antiepileptic that acts by binding to the synaptic vesicle protein SVA2, interfering with neurotransmitter exocytosis, and via inhibition of N-type calcium channels; it also increases tissue concentrations of GABA (162). Studies on the efficacy of levetiracetam in psychiatric disorders are relatively limited. There is some evidence that suggests potential benefit in the setting of treatment-refractory PTSD (120), and a small case series involving patients with alcohol dependence found improvement of anxiety symptoms with levetiracetam use (163). An open-label, flexible-dose study of levetiracetam (starting at 250 mg per day, up to 1500 mg twice per day) included 20 patients with SAD, and found a clinically significant 20.5-point decrease in Liebowitz Social Anxiety Scale (LSAS) scores in the intent-to-treat, last-observation-carried forward analysis; only 13 patients remained at the 8-week endpoint, with attrition attributed to side effects (*n* = 3), lack of efficacy (*n* = 1), and loss of follow-up (*n* = 3) (109). In a 7-week double-blind placebo controlled study involving 18 patients, 44% of the levetiracetam group showed response based on BSPS scores compared to 14% improvement in the placebo group levetiracetam, though results were not statistically significant using last-observation-carried-forward analysis; higher powered RCTs need to be conducted to gain a more decisive understanding of the utility of levetiracetam as a potential treatment for SAD (110). For generalized anxiety disorder, one 12-week double-blind randomized controlled trial of levetiracetam (starting dose 250 mg per day, flexibly titrated up to 1500 mg twice per day) involving 217 adult outpatients failed to demonstrate differences compared to placebo, with mean reductions in LSAS scores and response rates using levetiracetam equaling 24.4 and 41.3% respectively, and placebo demonstrating 28.7 point reductions and 46.6% response rate (111).

### Gabapentinoids

Although gabapentin and pregabalin are chemically similar to GABA, they actually bind and inhibit central nervous system voltage-gated α_2_-δ calcium channels and do not act via GABA receptors; this mechanism is thought to contribute to antiepileptic, antinociceptive, and anxiolytic effects (164). Notably, pregabalin exhibits greater potency as an α_2_-δ ligand compared to gabapentin, and it is also absorbed more rapidly and has greater bioavailability across dosing ranges.

Pregabalin has consistently been seen to improve anxiety symptoms among patients with GAD and has been effective both as monotherapy and as adjunct therapy (164, 165), and a meta-analysis from 2016 including 8 studies found pregabalin to be significantly superior to placebo in reducing HAM-A scores among patients with GAD (166). One early double blinded trial evaluated the effect of pregabalin at doses of 150 and 600 mg, lorazepam at 6 mg, and placebo on patients with GAD over a ten-week period; the drop in HAM-A scores seen in both the pregabalin low dose (-9.2) and high dose (-10.3) groups were comparable to the lorazepam group (-12.0) and significantly higher than the placebo (113). Follow up randomized control trials have shown that pregabalin has higher compliance rates than benzodiazepines therefore implying better tolerance of the drug. In addition, its efficacy was similar to the two to three times daily doses of benzodiazepines (112, 114).

Another double-blind trial compared the effects of pregabalin at 400 mg/d and 600 mg/d, the SNRI venlafaxine at 75 mg/d and a placebo on GAD. Both pregabalin groups showed an equivalent reduction in HAM-A scores with respect to the venlafaxine group. Pregabalin however had a faster onset of action of within 1 week (compared to the 2 weeks of the venlafaxine) and a much higher compliance rate (94–87% vs. 80% for venlafaxine). This implies that pregabalin may be preferable to SNRIs in patients with GAD (115). As an adjunctive therapy in patients with partial response to SSRIs or SNRIs, one randomized, double-blind, placebo-controlled trial showed superior efficacy of pregabalin (150–600 mg/d) compared to placebo after 8 weeks of combination treatment as demonstrated by statistically greater reduction in HAM-A scores (-7.6 vs. 6.4) and response rates (47.5 vs. 35.2%) (167).

The evidence for the efficacy of gabapentin is less robust and clinical trials are limited; one study found improvement in anxiety among breast cancer survivors (118), another in social phobia symptoms (116), while a third trial found gabapentin to more beneficial than placebo only among more severely affected patients and not in milder anxiety (117).

### Tiagabine

Tiagabine is an anti-epileptic drug, that selectively inhibits GABA reuptake, thereby decreasing glutamatergic activity. Common adverse effects seen in trials include dizziness, asthenia, somnolence, accidental injury, infection, headache, nausea, and nervousness. These were usually mild to moderate in severity and did not need medical intervention (168).

The largest tiagabine anxiety trial was an eight-week randomized placebo-controlled trial, on 266 subjects with GAD. The tiagabine treatment of upto 16 mg/d was associated with significantly lower HAM- A scores compared to placebo (119). An open-label trial with 54 patients demonstrated improvement in the Lieboweitz social anxiety scale, social phobia inventory and Sheehan Disability Scale after being administered 4-16 mg/d for 12 weeks (169).

Other trials on the use of tiagabine in anxiety disorders have been largely underpowered due to small sample sizes or lack of placebo groups or blinding. One open label trial on 28 subjects with panic disorder found statistically but not clinically significant reductions in panic disorder scales and HAM-A when they were administered 2-20 mg/day for 10 weeks (170). A later four-week RCT was conducted on 19 panic disorder patients in which the tiagabine group was started at 5 mg/d which was increased to a maximum of 30 mg/d depending on adverse effects. They found no difference between the tiagabine group and the placebo group in the clinical ratings. However, they also conducted panic challenges with CCK-4 in which the tiagabine treated subjects showed decreased sensitivity to experimentally induced panic (171). These mixed results may be disentangled by running larger studies that are better powered to pick up any possible effect. Another eight week, open-label study investigated the utility of tiagabine as an augmentation therapy for patients with anxiety that remained symptomatic despite appropriate anxiety drug trials. Tiagabine was started at 4 mg/d and flexibly increased to a maximum of 20 mg/d to optimize efficacy and tolerability, with a resulting mean dose of 13 mg/d. A response was reflected in the HAM-A scores were seen in thirteen (76%) of the subjects. This implies that patients who do not respond to the traditional first and second line anxiety may respond to tiagabine augmentation. However, this study only had eighteen participants and more adequate RCTs need to be conducted to confirm the results (172).

### Topiramate

Topiramate is an anti-epileptic that is also FDA approved as a migraine prophylactic. It has several mechanisms through which it alters neuronal function. It regulates Na^+^ and Ca^2+^ channel opening, potentiates GABA, and acts as an antagonist on AMPA and kainate receptors (173).

The results of a sixteen-week, open label trial with 23 subjects suggests its efficacy in the treatment of SAD. A flexible dose ranging from 25 to 75 mg/d was given to the active group. Seventy five percent of patients in the active group showed reduction in Leibowitz Social Anxiety Scale scores (174). Further, double-blind placebo-controlled trials are needed to determine the true efficacy of this agent.

### Valproic Acid

Valproic acid is believed to exert its antiepileptic effects by reducing neuronal activity via inhibition of voltage-gated ion channels, as well as by increasing concentrations of GABA levels by reducing its degradation (175).

Its effectiveness within anxiety disorders has not been thoroughly explored, and clinical trials are lacking. A six week RCT with eighty GAD patients found significantly more treatment responders (i.e., at least 50% improvement in Hamilton Anxiety Scale scores) and significantly greater symptom improvement (*p* < 0.001) among the group receiving 1500 mg/day of valproic acid compared to placebo (121). Benefits have also been reported in social anxiety disorder according to a 12 week open label flexible dose (500–2500 mg/day) study which showed significantly greater improvement in the Liebowitz Social Anxiety Scale (LSAS) in the treatment group (120). Another similar flexible dose open label study was conducted on ten participants with panic disorder. After a week-long placebo wash out period patients were given an ascending dose of valproic acid starting at 500 mg to a maximum of 2250 mg resulting in a significant improvement (*p* < 0.05) in the panic factor of the Symptom Checklist (SCL-90) (176). However, both these studies had a small sample size and a placebo lead in instead of a placebo group, thus, higher powered placebo-controlled trials would be needed to confirm these findings.

## Conclusion

Despite the presence of effective pharmacological and behavioral treatments, anxiety disorders remain a significant source of morbidity for many patients across the lifespan. Currently evidence-based pharmacological agents available to treat anxiety disorders include serotinergic antidepressants and benzodiazepines. Serotonergic antidepressants have a delayed onset of action, are only partially effective, leaving a substantial portion of patients unimproved or with significant residual symptoms after treatment, and have side-effects that somewhat limit adherence and tolerability. Benzodiazepines, although fast-acting and effective in treating anxiety symptoms, are not ideal because of the potential for patients to build tolerance or develop dependence with regular long-term use and also possible side-effects. Psychotherapy for anxiety disorder, although also quite effective, presents significant obstacles in terms of dissemination, access and acceptability to many patients. Thus, novel treatments for anxiety disorders are needed.

Drugs altering the glutamate and GABA systems have been increasingly studied as potential novel treatments for anxiety disorders. Evidence that abnormalities in the glutamate system are inherent to the pathophysiology of anxiety and other related-disorders strengths the rationale for studying these agents in clinical trials. Although breakthroughs have emerged regarding the benefits of glutamatergic agents such as ketamine and esketamine in the treatment of comorbid disorders such as Major Depression and possibly PTSD, strong evidence regarding the efficacy of glutamate modulating agents for anxiety disorders is currently lacking. Evidence regarding the efficacy of glutamatergic agents for anxiety disorder have largely been confined to uncontrolled and single-site studies. Large, adequately-powered, multi-site studies examining the efficacy of agents affecting glutamatergic neurotransmission in anxiety disorders are lacking and are needed.

## Author Contributions

The order of the author list reflects the amount of effort per person with MB and JD as senior authors.

## Conflict of Interest

MB receives research support from Therapix Biosciences, Neurocrine Biosciences, Janssen Pharmacueticals, Emalex Pharmaceuticals and Biohaven Pharmaceuticals. MB gratefully acknowledges additional research support from NIH, NARSAD, and the Patterson Foundation. The remaining authors declare that the research was conducted in the absence of any commercial or financial relationships that could be construed as a potential conflict of interest.
